# A comparison of anastomosis strength between sutures, staples, and self-forming magnets

**DOI:** 10.1016/j.igie.2022.10.007

**Published:** 2022-11-03

**Authors:** Derin Gumustop, Dane Seddon, Bora Gumustop, Jiping Wang

**Affiliations:** 1 Tufts University, Medford, Massachusetts, USA; 2 Department of Gastroenterology, St. Peter’s Hospital, Albany, New York, USA; 3 Division of Surgical Oncology, Brigham and Women’s Hospital, Harvard Medical School, Boston, Massachusetts, USA

## Abstract

Video 1

GI tract anastomoses are common procedures in abdominal surgeries. The current standard for anastomosis formation is the use of sutures by hand-sewn techniques or surgical staples. Creating GI anastomoses with surgical staplers has been the most popular technique that delivers dependable staple lines with consistent quality and rare technical failures.^1^ Staplers also provide operational advantages such as speed of performance, automation, and reproducibility.^2^, ^3^, ^4^ However, both hand-sewn and surgical stapling procedures involve penetrating tissue, thus invoking inflammation and damage to the surrounding tissue, which can cause leaks at the anastomotic site and other adverse events. Neither technique has yet been proved to be superior to the other, inasmuch as a meta-analysis found no difference in mortality, clinical leak rates, or cancer recurrence rates.^5^ Patients in groups undergoing stapling have also demonstrated a higher tendency toward stricture formation.^6^

As briefly mentioned, several severe and relevant adverse events are directly related to the stapled and hand-sewn anastomoses, including anastomotic leaks (ALs), with a frequency ranging from 1.8% to 19.2% depending on the site of anastomosis.^7^ Leakages lead not only to considerably higher risks of morbidity and mortality but also to an increased risk of reoperation. In some cases, overall mortality in patients has increased from 3.7% to 14.3% because of anastomotic leakage.^8^ As for the anastomotic healing process, ALs are likely to occur during the first 5 to 10 postoperative days when anastomotic healing is in its earliest stages. It has been shown that within 5 days of natural wound healing, the new tissue reaches a strength comparable with that of native tissue.^9^, ^10^, ^11^ This is further supported by findings that showed an increase of burst pressure reaching 60% of the strength of the surrounding bowel in 3 to 4 days and reaching equal burst pressures by 1 week.^12^ Thus, increasing the strength of the anastomosis to the same levels of surrounding healthy tissue as quickly as possible would be beneficial for the patient and decrease the risk of AL. Improving the safety and efficacy of anastomosis formation is still challenging, and alternatives to hand-sewn and stapled anastomoses could help reduce AL.

Magnetic compression anastomosis (MCA) is an evolving surgical method first demonstrated in 1978 by Obora et al.^13^ The process uses dynamic magnetic compression to compress tissue between 2 magnets and form an anastomosis. As the magnets move through tissue, the force between the 2 magnets will increase as they move closer to each other. Given that the process uses a noncontact force, it does not require any penetration of the tissue with sutures or staples.^14^ This technique has been successfully applied to a variety of anastomoses, including vascular anastomoses^15^ and GI anastomoses.^16^ This may prove advantageous for MCAs and their strength over hand-sewn and stapled techniques that do not increase compressive forces over time. To the author’s knowledge, the strength of an MCA has not been compared with those of hand-sewn and stapled anastomoses directly after anastomotic formation before wound healing, when ALs are most likely. This study aimed to use a novel self-forming MCA device and methodology for the construction of an anastomosis by using porcine intestine to compare its burst pressure during the initial time after formation with standard technologies and native tissue.

## Methods

### Study groups

The porcine small intestine was obtained from a commercial laboratory (Animal Technologies Inc, Tyler, Tex, USA). The intestinal tract was divided into 10-inch segments for each anastomosis formation. The intestinal segments were then distributed to 7 different study groups according to different anastomosis formation techniques:

Group 1 (n = 8): hand-sewn (HS)
Group 2 (n = 10): end-to-end staple
Group 3 (n = 6): side-to-side staple
Group 4 (n = 5): magnetic with 5 minutes of compression
Group 5 (n = 4): magnetic with 30 minutes of compression
Group 6 (n = 4): magnetic with 60 minutes of compression
Group 7 (n = 10): native tissue control group

The sample size was not uniform across groups owing to limited materials for each anastomosis technique.

### Anastomosis formation

#### Hand-sewn group

Double-layer anastomosis procedures were performed with the use of Ethicon 3-0 polydioxanone sutures on the inside layer in the running fashion and silk sutures on the outside layer in the interrupted fashion.

#### End-to-end stapled group

The anastomosis was performed with the Covidien EEA21 single-use circular stapler (Covidien, Minneapolis, Minn, USA) with directional stapling technology (DST) technology.

#### Side-to-side stapled group

**Figure 1:**
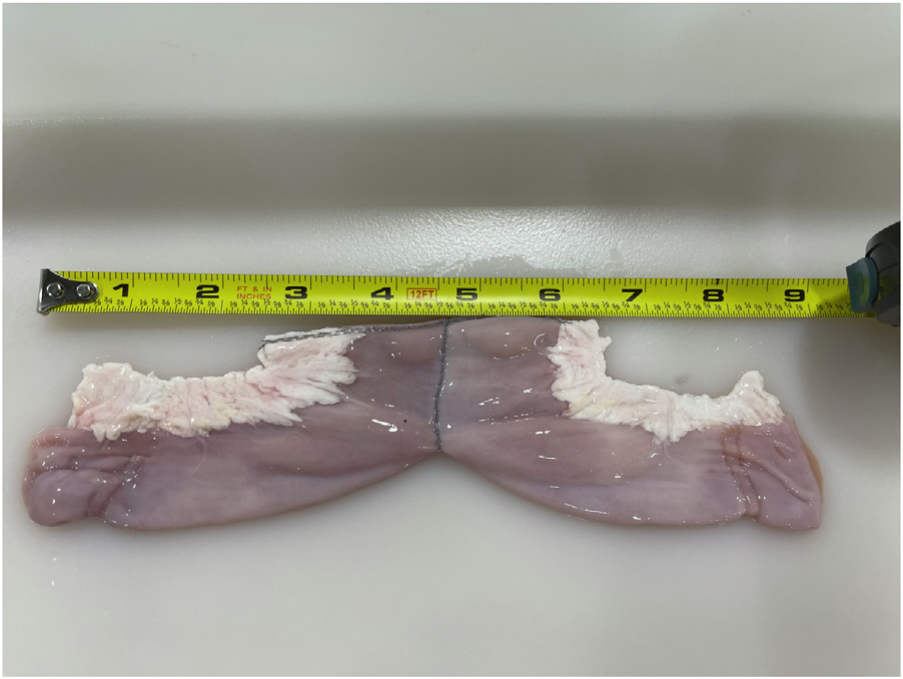
Side-to-side anastomosis.

Two linear staplers were used to perform a side-to-side anastomosis (Fig. 1).

#### Magnet compression groups

**Figure 2:**
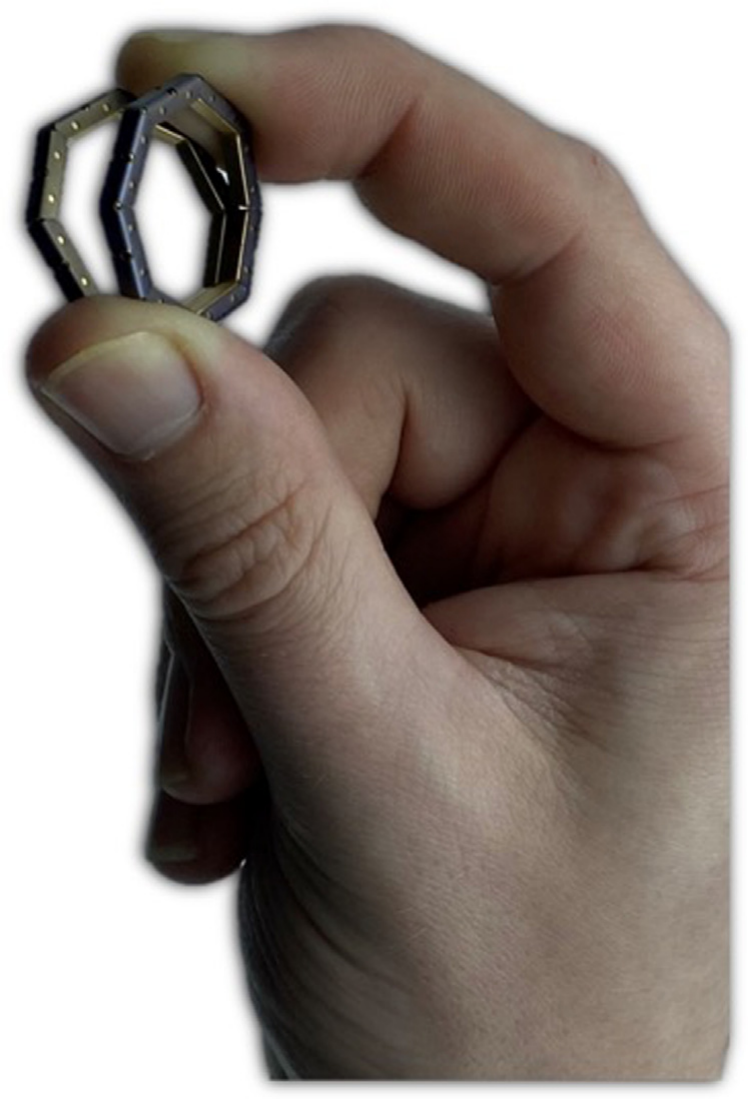
Self-forming magnets.

**Figure 3:**
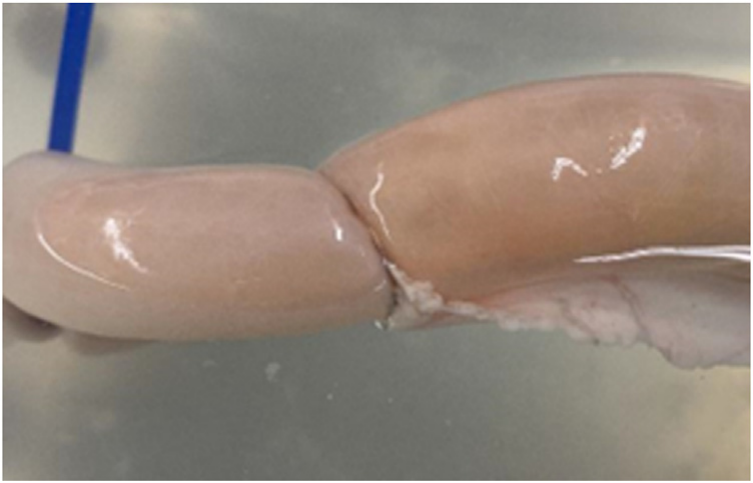
Magnetic compression anastomoses with intraluminal pressure.

A purse-string tool was used at the end of each intestinal lumen for tissue manipulation and coupling the self-forming magnets (SFMs). The SFM comprises 8 octagonal ring-shaped magnetic segments composed of gold and parylene-coated with an outer diameter of 25 mm. The SFMs were inserted into each luminal bowel end and maneuvered adjacent to the purse-string ends. The purse-string ends were pulled taut, centering the lumen with the SFMs. The respective SFMs were then brought together by use of a “heel-and-toe” method in which the bottom segments of the magnets were connected, followed by the top segments. The magnets were then left to compress the intestinal tissue for 5, 30, and 60 minutes, respectively (Figs. 2 and 3), before the burst pressure was tested.

### Measurement of anastomosis bursting pressure

**Figure 4:**
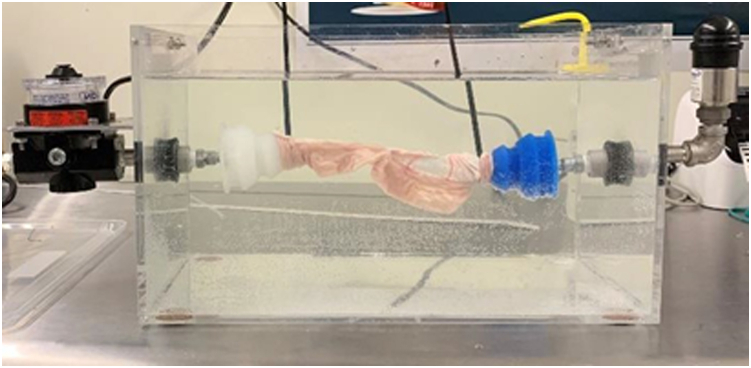
Burst pressure testing fixture.

For this experiment, a burst-pressure testing fixture was made (Fig. 4). The distal ends of the anastomotic intestine segments were slid over the silicone nozzles and tightly secured with zip ties. A compressed air source was then connected to the high-flow precision air regulator. The pressure gauge transducer was connected to the Pressure Pro application (Transducers Direct, Cincinnati, OH, USA) on an iPhone. Once the intestine was secured, the tank was filled up with water. Data collection was then started on the Pressure Pro application, and the air regulator was slowly opened, allowing air into the system and filling the intestine. The anastomosis was monitored until the first air bubbles were observed escaping from the anastomotic line (Video 1, available online at www.igiejournal.org). This was recorded as the bursting pressure and corresponded with the peak pressure recorded on the Pressure Pro application.

### Statistical analysis

SPSS statistics software v26.0 (IBM, Armonk, NY, USA) was used for data analysis. Quantitative data were expressed as a mean ± standard deviation (SD). Statistical significance was analyzed with the Mann-Whitney *U* test for comparison between groups . *P* < .05 indicated a significant difference.

## Results

**Figure 5:**
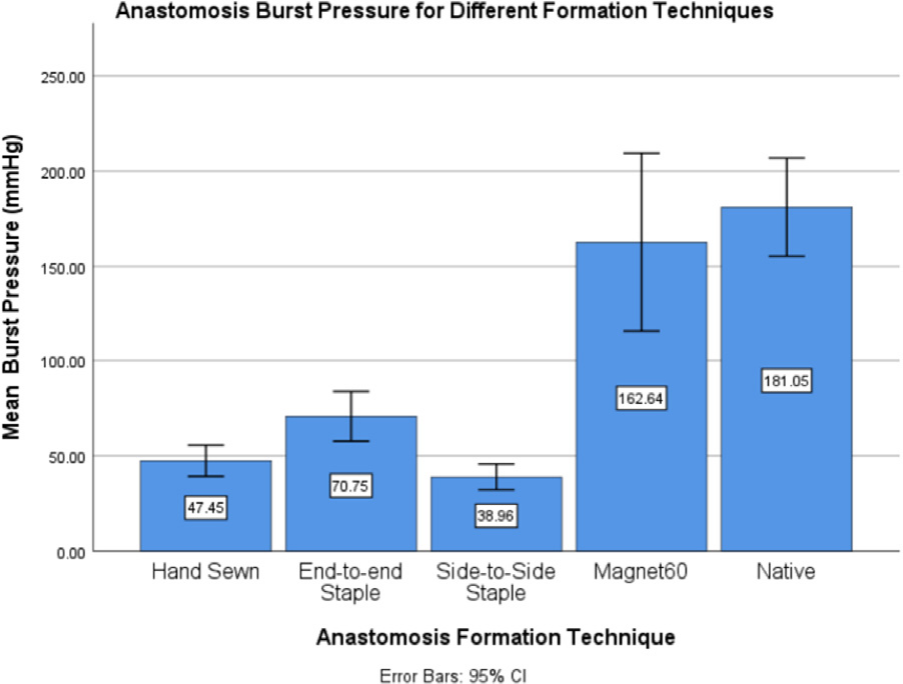
Mean anastomotic bursting pressures for magnetic compression anastomosis with different times of compression.

Anastomoses were successfully created for all porcine intestine segments. The MCAs were divided into 3 groups by the amount of time compressing the tissue: 5 minutes, 30 minutes, and 60 minutes. The effects of the constant compressive magnetic force were then evaluated across these different groups. The mean burst pressure (intraluminal pressure at failure) was 72.30 ± 16.06 mm Hg for the 5-minute group and 100.97 ± 13.14 mm Hg for the 30-minute group (*P* = .063) (Fig. 5). The burst pressure further increased to 162.64 ± 29.38 mm Hg for the 60-minute group and was statistically significant to both the 30-minute group (*P* = .029) and the 5-minute group (*P* = .016) (Fig. 5).

The hand-sewn group yielded a mean burst pressure of 47.45 ± 9.77 mm Hg, the side-to-side stapled group yielded the lowest bursting pressure of 38.96 ± 6.41 mm Hg, the end-to-end stapled group yielded 70.75 ± 18.30 mm Hg, and last the control group of native tissue yielded a burst pressure of 181.05 ± 36.02 mm Hg. The burst pressure averages and statistical significance between these groups are summarized in Table 1.

**Table 1:** Average anastomotic bursting pressures

Groups	Bursting pressure, mm Hg (mean ± SD)
Hand sutured	47.45 ± 9.77^a^
End-to-end stapled	70.75 ± 18.30^b^
Side-to-side stapled	38.96 ± 6.41^a^
Magnetic 5	72.30 ± 16.06^b,c^
Magnetic 30	100.97± 13.14^c^
Magnetic 60	162.64± 29.38^d^
Native tissue	181.05± 36.02^d^

Statistical difference between groups with different superscript letters: *P* < .05.

**Figure 6:**
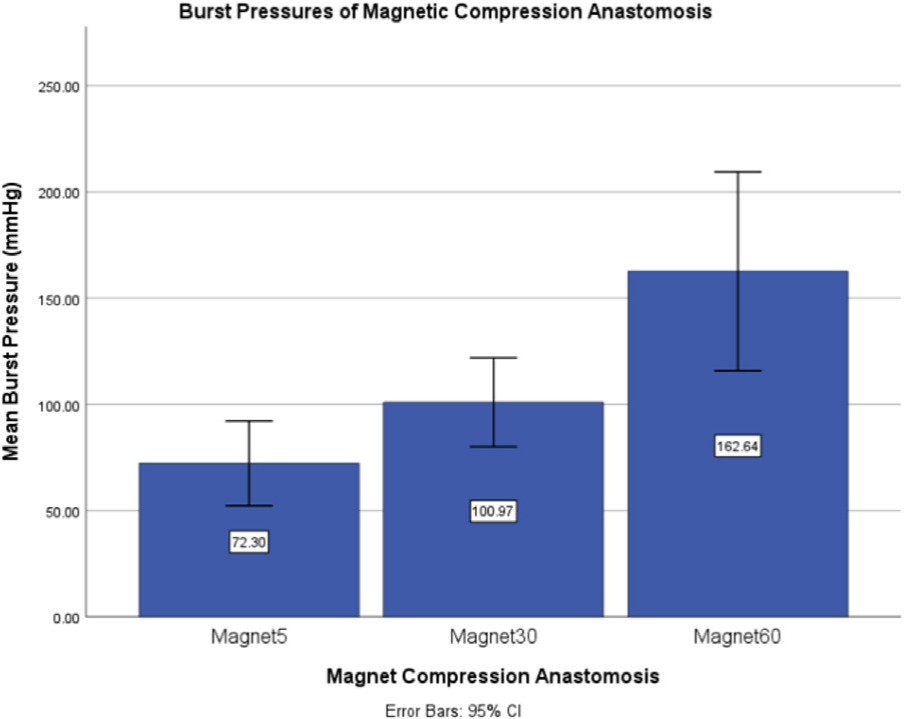
Comparison of mean burst pressure for 60-minute magnetic compression anastomosis to native tissue and standard technique.

After analysis of the differences between groups, it is notable that the end-to-end anastomosis was different from the hand-sewn anastomosis and side-to-side anastomosis. Also, once the MCA reached 30 minutes and 60 minutes, the burst pressures were different from hand-sewn and stapled anastomosis. A prominent result was that the MCA after 60 minutes of compression was not significantly different from that of the native tissue (*P* = .539). The burst pressure results from the 60-minute MCA are very promising and are compared with those of the native tissue and other standard techniques in Figure 6.

## Discussion

In summary, this ex vivo study compared burst pressures of hand-sutured, stapled, and magnetic anastomoses. The results indicated that the burst pressure of MCAs was significantly higher than those of hand-sewn and stapled anastomoses. By the end of 60 minutes after the anastomosis formation via magnetic compression, burst pressures were very close to that of native tissue (162 vs 181).

The results of this study indicated several key ideas. Given that burst pressure is an objective gauge to evaluate the relative strength of an anastomosis, it was observed that the strength of the MCA increased quickly with time. After the magnets were coupled, the anastomosis was created. By 30 minutes of compression, the MCA was significantly stronger than hand-sewn and stapler techniques. By 60 minutes of compression, the MCA was more than 3 times stronger than the hand-sewn group and more than 2 times stronger than the end-to-end stapled group. Additionally, by 60 minutes of compression, the MCA reached comparable strength of native tissue with no statistically significant difference in burst pressures.

A limitation of this study was the use of tissue explant. This ex vivo experimental study did not simulate actual magnetic anastomosis formation. There was no tissue necrosis due to ischemia, nor was new tissue formed. However, a strength of the study was the rigorous systematic comparison of the strengths of anastomoses immediately after formation, when necrosis and tissue healing is limited. Future studies will need to investigate in vivo the effectiveness of the self-forming magnets and the hypothesis that they will lower the risk of leaks.

## Disclosure

*The following authors disclosed financial relationships: D. Seddon: employee of GI Windows Surgical. J. Wang: consultant for GI Windows Surgical. B. Gumustop: Shareholder of GI Windows Surgical All other authors disclosed no financial relationships*.

## Supplementary data

Video 1 Anastomosis burst pressure fixture setup.
